# Biological Monitoring as a Preventive Occupational Healthcare Tool: Urinary Biomarkers of Benzene and Toluene Exposure Among Small-Scale Printing Workers in South Korea

**DOI:** 10.3390/healthcare14131856

**Published:** 2026-06-25

**Authors:** Jungho Hwang, Yangwoo Kim, Inah Kim, Seoyeon Kim, Juyeoung Hwang, Hyein Park, Ki-Youn Kim

**Affiliations:** 1 Korea Occupational Disease Surveillance Center, Hanyang University Seoul Hospital, Seoul 04763, Republic of Korea; schdom@gmail.com (J.H.); inahkim@hanyang.ac.kr (I.K.); seyon9611@gmail.com (S.K.); hgjuyg4226@naver.com (J.H.); phi8865@naver.com (H.P.); 2 Graduate School of Public Health, Hanyang University, Seoul 04763, Republic of Korea; 3 Graduate School of Safety Engineering, Seoul National University of Science and Technology, Seoul 01811, Republic of Korea; 4 Department of Occupational and Environmental Medicine, Inha University Hospital, Incheon 22332, Republic of Korea; oem.ywkim@gmail.com; 5 Department of Social and Preventive Medicine, Inha University College of Medicine, Incheon 22332, Republic of Korea; 6 Department of Safety Engineering, Seoul National University of Science and Technology, Seoul 01811, Republic of Korea

**Keywords:** occupational health surveillance, preventive occupational healthcare, biological monitoring, urinary biomarkers, benzene, toluene, organic solvents, small enterprises, printing workers, occupational exposure assessment

## Abstract

**Highlights:**

**What are the main findings?**

- Urinary o-cresol levels exceeded the biological exposure index in 42.9% of small-scale printing workers.
- Reported benzol handling showed a suggestive association with elevated urinary SPMA levels.

**What are the implications of the main findings?**

- Biological monitoring may support preventive occupational healthcare in small enterprises.
- Local chemical handling terminology should be carefully considered when conducting occupational exposure surveillance in multilingual work environments.

**Abstract:**

**Background/Objectives**: Small-scale workplaces often have limited access to occupational health services, despite potential exposure to hazardous solvents. Biological monitoring can provide worker-level evidence of internal exposure when routine environmental monitoring is limited. This study evaluated urinary biomarkers of benzene and toluene exposure among workers in small-scale printing workplaces in South Korea and examined whether self-reported chemical handling corresponded with biomarker patterns. **Methods**: In this cross-sectional field biomonitoring study, 21 workers from eight printing companies provided end-of-shift urine samples. Creatinine-adjusted urinary concentrations of trans,trans-muconic acid (t,t-MA), S-phenylmercapturic acid (SPMA), phenol, and o-cresol were analyzed and compared with applicable biological reference values. Associations between reported chemical handling and elevated biomarker levels were estimated using Firth’s penalized logistic regression, and correlations among log-transformed biomarkers were explored. **Results**: Nine workers (42.9%) had urinary o-cresol concentrations at or above the American Conference of Governmental Industrial Hygienists (ACGIH) biological exposure index of 300 μg/g creatinine. Workers reporting benzol handling, a local term for petroleum-based cleaning products, had higher odds of elevated SPMA, although the estimate was imprecise and hypothesis-generating (age-adjusted OR 6.04, 95% CI 0.75–104.90, *p* = 0.093). The correlation between SPMA and t,t-MA was stronger among workers reporting benzol handling (r = 0.94) than among those reporting toluene handling (r = −0.01). **Conclusions**: These exploratory findings indicate that toluene-related internal exposure is a relevant occupational health concern in small-scale printing workplaces, while reported benzol handling may indicate possible low-level benzene-related exposure. Urinary biomonitoring may support exposure surveillance and preventive occupational healthcare in small enterprises when interpreted alongside workplace observations, product information, ventilation, protective equipment use, and worker education.

## 1. Introduction

The printing industry includes many small, labor-intensive workplaces located in dense, urban commercial districts [1]. Printing processes are associated with recognized occupational hazards, including exposure to volatile organic compounds and carcinogenic agents in inks, thinners, and cleaning products [2,3,4]. In South Korea, these concerns are particularly relevant because many printing businesses operate on a small scale and have limited capacity for systematic occupational health management [5]. Organic solvents are commonly handled during printing, plate cleaning, and equipment maintenance, whereas routine environmental exposure monitoring may be infrequent in small enterprises [6,7].

From a healthcare perspective, workers in small enterprises represent a population that may be underserved by conventional occupational health services [5,8]. Limited access to routine exposure assessments, occupational health consultations, and preventive monitoring can delay the recognition of hazardous internal exposure [8,9]. Therefore, field-based biomonitoring may function not only as an exposure assessment method but also as a preventive occupational healthcare strategy for identifying workers and workplaces that require targeted interventions [10,11].

Benzene and toluene are important aromatic hydrocarbons in this setting. Benzene is a well-established human carcinogen associated with hematological malignancies, and even low-level occupational exposure remains a concern in solvent-using workplaces [12]. Toluene is less hematotoxic than benzene but is widely used in industrial solvent mixtures and is associated with neurotoxic effects after repeated exposure [13]. In Korean printing workplaces, workers may use the term “benzol” to describe petroleum-based cleaning products rather than pure benzene. This local terminology poses a practical challenge for exposure assessment because the reported product category may encompass mixtures containing benzene, toluene, and other hydrocarbons.

Biological monitoring can complement air sampling by capturing internal exposure integrated across tasks, uptake routes, and short-term variations in work practices [14,15]. Urinary S-phenylmercapturic acid (SPMA) and trans,trans-muconic acid (t,t-MA) are established biomarkers of benzene exposure, although SPMA is generally considered more specific at low exposure levels. Urinary phenol has also been used historically for benzene exposure assessment, but its specificity is limited under contemporary low-level benzene exposure. For toluene, urinary o-cresol is an established biological indicator that can be used to evaluate internal exposure [13,16,17].

Previous studies of printing workplaces have assessed benzene concentrations in petroleum-based cleaning products and workplace air samples; however, these field studies relied on area sampling and noted the need for personal-level exposure assessment [6,18]. Individual biomonitoring data on workers in small-scale Korean printing workplaces are limited. Such data may help determine whether questionnaire-reported handling of solvent products corresponds to measurable internal exposure.

This study evaluated the urinary biomarkers of benzene and toluene exposure among workers in small-scale printing workplaces in South Korea. We also examined whether self-reported chemical handling was associated with elevated biomarker concentrations and explored whether correlations among urinary biomarkers differed according to the type of chemical handled by workers. Beyond characterizing internal exposure, we aimed to assess whether biological monitoring could provide actionable information for occupational health surveillance in small-scale enterprises where routine environmental monitoring and systematic health management may be limited.

## 2. Materials and Methods

### 2.1. Study Design and Setting

This cross-sectional field biomonitoring study was conducted among workers in small-scale printing workplaces in the Seoul Metropolitan Area, Republic of Korea. Participants were recruited in 2025 through the High-Risk Industrial Health Comprehensive Management Program. The program targeted workplaces in printing and cleaning processes that were designated as regionally specialized sectors under the occupational health management program.

Of the 111 eligible workplaces, eight companies agreed to participate through collaboration between the Korea Occupational Disease Surveillance Center (KODSC) [19,20] and the Seoul Regional Headquarters of the Korea Occupational Safety and Health Agency (KOSHA). This study was designed to evaluate internal exposure to aromatic hydrocarbon solvents using urinary biomarkers in workers. This study also examined whether questionnaire-reported chemical handling was associated with elevated biomarker concentrations. This recruitment framework reflects a real-world occupational health surveillance setting in which public occupational health institutions identify high-risk small enterprises and provide field-based assessment. Because participation was voluntary, participating workplaces may have differed systematically from non-participating workplaces in occupational health awareness, solvent use patterns, ventilation conditions, or willingness to cooperate with public occupational health programs, which may limit the generalizability of the estimates reported here.

### 2.2. Participants

Workers were eligible if they were employed in a participating printing workplace and were currently engaged in printing- or cleaning-related tasks. The study protocol specified exclusion criteria of a history of urological malignancy and physical limitations affecting urine sample collection; no enrolled workers met these criteria. Workers were excluded from the biomarker analysis if they did not complete the questionnaire or if urinary biomarker data were unavailable.

A total of 26 workers were initially recruited. Two non-Korean workers were excluded before the survey dataset was constructed because they were unavailable during the workplace visit and did not complete the questionnaire. Therefore, the survey dataset included 24 workers. Three additional workers were excluded because end-of-shift urine samples were not submitted and urinary biomarker data could not be obtained. The final analytical sample included 21 workers from eight worksites.

Written informed consent was obtained from all participants before enrollment. The study protocol was approved by the Institutional Review Board of Hanyang University (IRB No. HYUIRB-202509-026, approved on 16 September 2025). Participants were selected following the study’s approval, and all study procedures were conducted in accordance with the principles of the Declaration of Helsinki.

### 2.3. Questionnaire and Workplace Assessment

A structured questionnaire was administered to collect information on demographic characteristics, work history, job tasks, chemical handling, product use frequency, occupational health management, lifestyle factors, chronic diseases, self-reported health status, and symptoms. Multiple responses were allowed for job tasks and chemical handling items. Translated versions of the questionnaire were prepared for non-Korean workers.

Chemical handling variables were based on self-reported handling of benzol, toluene, mixed solvents, alcohol, water-based products, and ink. In this manuscript, “benzol” denotes the questionnaire exposure category used by workers to refer to petroleum-based cleaning products that may contain benzene [21]. Multiple responses were allowed for chemical handling items. To minimize the potential for misunderstanding, the questionnaire was administered in a structured interview format by trained occupational health professionals. To support internal cross-checking, workers were asked separately about their primary duties, frequently performed tasks, and the types of chemicals used for each task. The term benzene was used only when referring to the chemical agent or benzene-related biomarker interpretation. Although this structured approach was intended to reduce inaccurate self-reporting, self-reporting bias could not be fully eliminated. The standard questionnaire is provided in Supplementary File S1.

Workplace-level information was collected during site visits and from workplace representatives when the individual respondents could not provide information. Workplace variables included company size, local exhaust ventilation, provision of chemical-resistant gloves, and safety and health education.

These workplace-level indicators were selected because they represent the fundamental components of preventive occupational health management in small enterprises, including engineering controls, personal protective equipment provision, and worker health education.

### 2.4. Urinary Biomarkers

Spot urine samples were collected at the end of the work shift. Four urinary biomarkers were analyzed: trans,trans-muconic acid (t,t-MA), S-phenylmercapturic acid (SPMA), phenol, and o-cresol. t,t-MA, SPMA, and phenol were considered benzene-related biomarkers [15], whereas o-cresol was considered a toluene-related biomarker [13].

Urinary t,t-MA and SPMA were quantified using liquid chromatography–tandem mass spectrometry (LC-MS/MS; Triple Quad 5500+ System, AB Sciex LLC, Marlborough, MA, USA), and urinary phenol and o-cresol were quantified using headspace gas chromatography–mass spectrometry (HS-GC-MS; Clarus 680 GC coupled with a Clarus SQ 8T mass spectrometer, PerkinElmer, Shelton, CT, USA). Urinary creatinine was measured using a kinetic colorimetric assay based on the Jaffe method. The limits of detection for t,t-MA, SPMA, phenol, and o-cresol were 0.580 μg/L, 0.030 μg/L, 0.066 mg/L, and 0.006 mg/L, respectively. All biomarker concentrations were corrected for urinary creatinine and expressed as micrograms per gram creatinine. Additional details on sample preparation, internal standards, quality-control materials, limits of detection, and limits of quantification are provided in Supplementary Table S2.

Biomarker concentrations were compared with the applicable biological reference values. The applied ACGIH biological exposure index (BEI) values were 500 µg/g creatinine for t,t-MA, 25 µg/g creatinine for SPMA, and 300 µg/g creatinine for o-cresol according to the ACGIH TLVs and BEIs [22]. Although urinary phenol is no longer recommended by the ACGIH as a benzene BEI owing to its limited specificity at low exposure levels, it was retained in this analysis because it is included in the Korean occupational exposure standards [23]. For benzene-related biomarkers with ACGIH BEI values, the concentrations were categorized into three levels: below 10% of the BEI, 10–99% of the BEI, and at or above the BEI. The same threshold structure was applied to the Korean phenol reference values for descriptive purposes. For o-cresol, the at-or-above BEI category was the primary threshold of interest for toluene exposure assessment.

For the exploratory analysis of elevated biomarker levels, each biomarker was categorized into quartiles, and the highest quartile was compared with the lower three quartiles. Quartile-based outcomes were preferred for regression analyses because BEI exceedance was rare for several biomarkers: most t,t-MA and SPMA concentrations were below the corresponding BEI values, and no participant exceeded the Korean biological reference value for phenol. Therefore, BEI exceedance was not suitable as a regression outcome for several exposure–biomarker combinations because it would have produced unstable or non-estimable estimates. The quartile-based outcome was used to identify relatively elevated biomarker levels within this exploratory field-surveillance sample, whereas BEI categories were retained as clinical and occupational health interpretive benchmarks.

### 2.5. Statistical Analysis

Descriptive statistics were calculated for worker- and workplace-level characteristics. Continuous variables are presented as mean and standard deviation or as geometric mean and geometric standard deviation, when appropriate. Categorical variables are presented as frequencies and percentages.

As SPMA is recommended by the ACGIH as a specific biomarker for low-level benzene exposure [22], participant characteristics were compared between workers in the highest quartile of SPMA and those in the lower three quartiles. Fisher’s exact test was used for categorical variables, and the independent-samples *t*-test was used for continuous variables. These comparisons were interpreted descriptively because of small sample sizes.

Because the analytic sample was small and several exposure-outcome combinations had sparse cells, associations between self-reported chemical handling and elevated biomarker levels were estimated using Firth’s penalized logistic regression [24,25]. Separate models were fitted for each biomarker highest-quartile outcome. Crude odds ratios and age-adjusted odds ratios were estimated with 95% confidence intervals. Age was selected for adjustment a priori because of its potential association with work history and biomarker levels. Smoking status was examined descriptively but was not included as a covariate in the primary adjusted models because the small sample size and sparse outcome counts would have produced unstable estimates. Therefore, the primary models were adjusted only for age. Because smoking can influence urinary biomarkers related to aromatic hydrocarbon exposure, residual confounding by smoking was considered when interpreting the results and is acknowledged as a limitation.

Pairwise correlations among log-transformed urinary biomarkers were examined using Pearson correlation coefficients. Correlation plots were generated to visualize the relationships between benzene-related biomarkers and the toluene-related biomarker.

All analyses were conducted using R version 4.5.1. Firth logistic regression was performed using the logistf package. version. 1.26.1 [26]. Statistical significance was defined as a two-sided *p*-value below 0.05. *p*-values from 0.05 to below 0.10 were described as suggestive evidence because this was an exploratory field study with a limited sample size. Given that four urinary biomarkers and two primary exposure categories were examined, some statistically noteworthy findings may reflect chance variation from multiple comparisons, and all results should be treated as hypothesis-generating pending replication in larger samples.

## 3. Results

### 3.1. Participant Characteristics

The demographic and occupational characteristics of the 21 participants are summarized in Table 1. The mean age was 45.6 ± 14.0 years, and all participants were male. Seventeen workers (81.0%) were Korean, and the remaining four were foreign nationals. The most common job types were offset printing (71.4%) and UV printing (42.9%); multiple responses were permitted. Nine workers (42.9%) reported handling benzol, a term used in Korean printing workplaces to refer to petroleum-based cleaning products that may contain benzene, and eight (38.1%) reported handling toluene. Because multiple responses were permitted, exposure categories overlapped: eight workers reported benzol only, seven reported toluene only, one reported both benzol and toluene, and five reported neither benzol nor toluene. Table 1 also presents the participant characteristics stratified by urinary SPMA quartile; except for a suggestive difference in educational level (*p* = 0.07), no characteristics differed significantly between the highest quartile (Q4) and the lower three quartiles (Q1–Q3). All 21 participants were male, which reflects the actual workforce composition of the 8 participating workplaces. Therefore, it may be difficult to generalize the study results to female workers or mixed-gender printing workplaces.

### 3.2. Workplace Characteristics

The eight participating workplaces included six medium-sized enterprises with 5 to 49 employees (75.0%), one small enterprise with fewer than 5 employees, and one with 50 or more employees. Local exhaust ventilation was present in three workplaces (37.5%), chemical-resistant gloves were provided in four workplaces (50.0%), and safety and health education was conducted in two workplaces (25.0%). These findings indicate that basic occupational health management measures were not uniformly implemented across the participating workplaces, despite the presence of solvent-related tasks in the work processes of the employees. The limited availability of local exhaust ventilation, chemical-resistant gloves, and safety and health education provides contextual information for interpreting urinary biomarker findings as part of a workplace health surveillance framework. Although one participating workplace had 50 or more employees, the study targeted the small-scale printing sector under a high-risk occupational health management program, and most participating workplaces had fewer than 50 employees.

### 3.3. Urinary Biomarker Concentrations

The most clinically notable finding was that nine workers (42.9%) had urinary o-cresol concentrations at or above the ACGIH BEI of 300 μg/g creatinine, indicating substantial toluene-related internal exposure in this cohort. Table 2 summarizes the urinary biomarker concentrations. The geometric means were 94.2 μg/g creatinine (GSD 3.55) for t,t-MA, 2.3 μg/g creatinine (GSD 2.47) for SPMA, 4926.9 μg/g creatinine (GSD 2.22) for phenol, and 320.6 μg/g creatinine (GSD 2.33) for o-cresol. Two workers (9.5%) had t,t-MA concentrations at or above the ACGIH BEI, and one worker (4.8%) had SPMA concentrations at or above the ACGIH BEI. None of the participants exceeded the Korean biological reference values for urinary phenol. In contrast, o-cresol showed the highest proportion of reference-value exceedance. Most participants had SPMA concentrations below 10% of the BEI (81.0%), whereas no participant had o-cresol concentrations below 10% of the BEI.

### 3.4. Association Between Chemical Handling and Elevated Biomarkers

Table 3 presents the associations between self-reported chemical handling and elevated biomarker levels, defined as the highest quartile, estimated using Firth’s penalized logistic regression. Workers who reported handling benzol had higher odds of belonging to the highest quartile of SPMA in both the crude analysis (OR 6.27, 95% CI 0.88–74.98, *p* = 0.068) and age-adjusted analysis (OR 6.04, 95% CI 0.75–104.90, *p* = 0.093), although the confidence intervals were wide. Age was associated with elevated t,t-MA levels in the crude model (OR 1.10 per 1-year increase, 95% CI 1.01–1.29, *p* = 0.034); however, similar associations were not observed for the other biomarkers. Reported benzol handling was not associated with elevated levels of t,t-MA, phenol, or o-cresol. Reported toluene handling was not significantly associated with elevated o-cresol levels, although the age-adjusted odds ratio was above 1 (OR 2.97, 95% CI 0.45–23.67, *p* = 0.256).

### 3.5. Correlations Among Urinary Biomarkers

**Figure 1:**
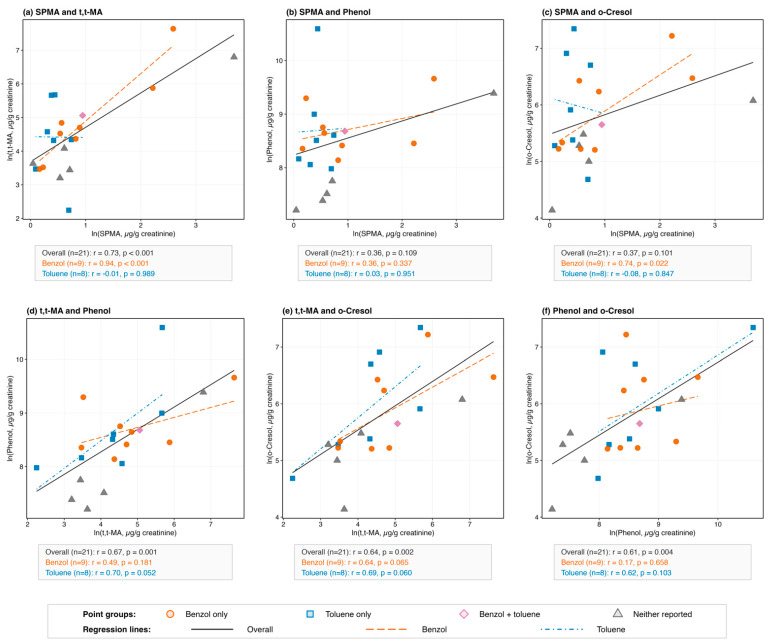
Pairwise correlations among log-transformed urinary biomarkers based on self-reported chemical handling.

Figure 1 shows the pairwise correlations among the log-transformed urinary biomarkers. The overall correlation between SPMA and t,t-MA was 0.73 and was particularly strong among workers who reported benzol handling (r = 0.94). By contrast, the correlation was near zero among workers who reported toluene handling (r = −0.01). The SPMA–o-cresol correlation was moderate in the overall sample (r = 0.37) and stronger in the benzol-handling subgroup (r = 0.74). Overall correlations involving o-cresol were moderate for t,t-MA (r = 0.64) and phenol (r = 0.61) but weaker for SPMA.

## 4. Discussion

### 4.1. Main Findings

This cross-sectional field biomonitoring study examined urinary biomarkers of benzene and toluene in 21 workers from eight small-scale printing workplaces in South Korea. Urinary o-cresol showed the highest proportion of BEI exceedance (42.9%), suggesting relevant toluene-related internal exposure in this worker group. Workers who reported handling benzol had higher odds of elevated SPMA levels; however, the confidence interval was wide and included values below 1.0 (age-adjusted OR 6.04, 95% CI 0.75–104.90, *p* = 0.093), and this result should be interpreted as hypothesis-generating rather than confirmatory. SPMA and t,t-MA were moderately correlated overall (r = 0.73), and this correlation was stronger among workers reporting benzol handling (r = 0.94) than among those reporting toluene handling (r = −0.01).

### 4.2. Interpretation of Benzene Biomarkers

The association between reported benzol handling and elevated SPMA is biologically plausible, even though it did not reach conventional statistical significance. SPMA is regarded as a relatively specific biomarker of benzene exposure, particularly under low-level occupational exposure [14,15]. The wide confidence interval is compatible with the small sample size and the small number of workers in the highest SPMA quartile. Therefore, the results should be interpreted as suggestive rather than confirmatory evidence of benzene-related internal exposure among workers who reported handling benzol. This interpretation is consistent with prior evidence that petroleum-based cleaning products used in Korean printing workplaces may contain benzene [6,21]. Specifically, because the lower bound of the confidence interval fell below 1.0, the direction of association remained uncertain, and the finding was best characterized as a signal warranting confirmation in larger studies.

The correlation analysis also supported cautious differentiation based on reported chemical handling. The strong SPMA–t,t-MA correlation among workers who reported benzol handling is consistent with a shared benzene-related exposure source. In contrast, the near-zero SPMA–t,t-MA correlation among workers reporting toluene handling suggests that the relationship between benzene biomarkers was not uniform across questionnaire-defined exposure groups. The stronger SPMA–o-cresol correlation in the benzol-handling subgroup may reflect mixed exposure to aromatic hydrocarbon solvents in petroleum-based cleaning products rather than a single-agent exposure pattern. Given the small subgroup sizes, these correlations should be viewed as exploratory signals that can help generate hypotheses for future biomonitoring studies.

These findings further illustrate the limitations of less specific benzene-related biomarkers. Although t,t-MA was correlated with SPMA in the overall sample and in the benzol-handling subgroup, it was not associated with reported benzol handling in the regression analysis. t,t-MA can be influenced by non-occupational factors, including dietary sorbic acid, and is less specific than SPMA under low-level exposure conditions [15,27]. Phenol showed no exceedance of the Korean biological reference value and was not associated with the reported exposure variables. This pattern is consistent with previous evidence that urinary phenol has limited discriminatory value for low-level occupational benzene exposure because of background and non-occupational sources of exposure [14,15]. Compared with previous studies of benzene exposure in printing or solvent-using workplaces, the benzene-related biomarker pattern in the present study appears to be more consistent with low-level or intermittent benzene exposure than with high-intensity benzene exposure. Previous studies in Korean printing workplaces reported that petroleum-based cleaning products and related work environments may contain benzene [6,18,21], but individual-level urinary biomonitoring data in this specific small-scale printing setting have been limited. In the present study, only one worker exceeded the ACGIH BEI for SPMA and two workers exceeded the BEI for t,t-MA, whereas no participant exceeded the Korean biological reference value for phenol. This pattern differs from what would be expected in settings with substantial benzene exposure and is more compatible with low-level benzene impurities in petroleum-based cleaners, intermittent cleaning tasks, and mixed-solvent use. Differences from other occupational settings may also reflect product formulation, ventilation conditions, use of protective equipment, timing of urine collection, and non-occupational influences such as smoking or dietary sorbic acid intake, particularly for t,t-MA and phenol [14,15,27]. Therefore, the benzene-related findings should be interpreted as a signal of possible low-level internal exposure rather than as evidence of widespread high-level benzene exposure.

### 4.3. Toluene Exposure and Interpretation of o-Cresol

The o-cresol findings deserve particular attention because toluene-containing solvents are frequently encountered in printing operations [7,13]. Reported toluene handling was not significantly associated with elevated o-cresol in the regression models, although the direction of association was positive (aOR 2.97, *p* = 0.256). This discrepancy may reflect imprecise self-reported classification of chemicals handled, mixed exposure across multiple products and tasks, or limited statistical power. This also underscores the inherent difficulty in attributing biomarker patterns to a single solvent in small workplaces, where product composition may be poorly characterized. It should also be noted that urinary o-cresol may be elevated by non-occupational sources, including dietary phenolic compounds and, to a lesser extent, smoking. Although elevated o-cresol in this occupational cohort likely reflects, at least in part, workplace toluene exposure, contributions from smoking (42.9% current smokers in this sample) and non-occupational exposures cannot be excluded, and BEI exceedance should be interpreted in the context of the worker’s full exposure history. The o-cresol pattern was more prominent than the benzene-related biomarker pattern and was broadly consistent with previous occupational biomonitoring studies of toluene-exposed printing or solvent-using workers. Urinary o-cresol has been evaluated as a biological indicator of toluene exposure in rotogravure printing and other toluene-exposed worker groups [13,16,17]. In the present study, 42.9% of participants had urinary o-cresol concentrations at or above the ACGIH BEI, whereas most workers had SPMA concentrations below 10% of the BEI. This contrast suggests that toluene-related internal exposure may have been more prominent than benzene-related exposure in this small-scale printing sample. However, direct quantitative comparison with previous studies should be made cautiously. Urinary o-cresol levels can vary considerably according to printing process, solvent formulation, intensity and duration of use, ventilation conditions, timing of urine collection relative to exposure, analytical method, and creatinine correction approach [13,16,17]. The absence of a statistically significant association between self-reported toluene handling and elevated o-cresol may also reflect local product terminology. Workers may report a solvent-containing product as “benzol,” thinner, or cleaner without specifying its chemical composition, even when the formulation contains toluene or other aromatic hydrocarbons [7,21].

From an occupational health perspective, these findings are relevant because small printing enterprises often face structural limitations in exposure surveillance [5]. In settings where routine air monitoring is infrequent or unavailable, biomonitoring can serve as a complementary tool for identifying internal exposure that may not be detected by occasional environmental measurements alone. More broadly, occupational exposure assessments have emphasized that legacy hazards may persist when historical product use and current control practices are incompletely documented [28]. The finding that o-cresol exceeded the BEI in nearly half of the participants, whereas most workers had SPMA concentrations below 10% of the BEI, suggests that benzene- and toluene-related exposure patterns may differ even within the same occupational setting.

### 4.4. Implications for Preventive Occupational Healthcare in Small Enterprises

The present findings have practical implications for occupational healthcare delivery in small-scale enterprises. Small printing workplaces may have limited capacity to conduct regular environmental monitoring, interpret safety data sheets, implement engineering controls, and provide systematic worker education and training. In these circumstances, biological monitoring can assist occupational health personnel in identifying employees with detectable internal exposure and determining where further workplace interventions are necessary to reduce exposure. The cost of urinary biomonitoring can be a practical barrier for small and medium-sized enterprises. When biomonitoring is required or recommended as part of occupational health surveillance, the financial burden should not be shifted to individual workers. Under the Occupational Safety and Health Act, employers are responsible for conducting special health examinations and pre-placement health examinations for workers assigned to tasks involving designated hazardous agents [29]. Public support mechanisms are also available for smaller workplaces. For example, KOSHA’s Small Workplace Health Stepping Stone Program supports work environment monitoring and pre-placement or special health examination costs for eligible small workplaces [30]. In addition, the Standards for Implementation of Workers’ Health Examinations provide a framework for government support or subsidy for all or part of the costs incurred for special and pre-placement health examinations [31]. These funding routes should be considered when targeted biomonitoring is recommended as part of preventive occupational health strategies for the small-enterprise sector.

The high proportion of workers with urinary o-cresol concentrations at or above the BEI suggests that toluene-related exposure should be addressed through a combination of product substitution, improved ventilation, appropriate glove use, worker education, and periodic health surveillance. The suggestive association between reported benzol handling and elevated SPMA levels also indicates that local chemical-handling terminology should be carefully considered in occupational health assessments. Workers may report product names or colloquial categories rather than specific chemical ingredients, which can lead to exposure misclassification unless biomonitoring or product composition data are used to confirm exposure.

These findings suggest that occupational health programs for small printing workplaces should prioritize sites where petroleum-based cleaning products or mixed solvents are used and integrate biological monitoring with workplace visits, product composition reviews, and follow-up consultations.

### 4.5. Limitations and Strengths

Several limitations should be considered. First, the cross-sectional design precludes causal inferences. Second, the sample size was small, resulting in wide confidence intervals, limited statistical power, and unstable subgroup correlations. Third, among the 111 qualified workplaces, only eight chose to participate, leading to a participation rate of 7.2%. This low participation rate may limit generalizability and may have introduced selection bias if participating workplaces were more health-conscious, more willing to cooperate with public occupational health programs, or different from non-participating workplaces in terms of solvent use, ventilation status, and occupational health practices. Therefore, the findings should be interpreted as exploratory evidence from a participating field-surveillance sample rather than as representative estimates for all small-scale printing workplaces.

Fourth, exposure classification relied on self-reported chemical handling and may be subject to misclassification; the benzol category, in particular, likely included products with varying aromatic hydrocarbon compositions. Fifth, spot urine samples collected at a single end-of-shift time point may not represent the long-term exposure variability. Sixth, environmental air monitoring and product composition data were not available for direct comparison with the urinary biomarker levels. Seventh, residual confounding by smoking, diet, and non-occupational solvent exposure cannot be excluded, particularly for t,t-MA, phenol, and o-cresol. Eighth, workers from the same printing workplace may share ventilation conditions, solvent products, and work practices, which could introduce within-workplace correlation in biomarker levels and reduce the statistical independence of observations in the regression analyses. With only eight workplaces, multilevel modeling was not feasible; therefore, the potential for within-workplace clustering represents an additional source of imprecision in the regression estimates. Finally, this study did not include a formal cost-effectiveness analysis; therefore, future studies should evaluate the economic feasibility, prioritization criteria, and sustainable financing models for biomonitoring programs in small enterprises.

These limitations are balanced by the strengths of this study. This study focused on small-scale printing workplaces, an understudied population in occupational exposure biomonitoring. Multiple urinary biomarkers were measured simultaneously, allowing for a comparison of benzene- and toluene-related exposure indicators. The study also linked biomarker results with questionnaire-based information on handled chemicals, which is practically relevant for occupational health programs serving small enterprises. At the same time, these limitations reflect the real-world constraints of small-enterprise occupational health surveillance and highlight the practical value of biological monitoring when product composition and routine air monitoring data are unavailable.

## 5. Conclusions

In conclusion, this exploratory field biomonitoring study found that 42.9% of workers had urinary o-cresol concentrations at or above the ACGIH BEI, indicating that toluene-related internal exposure is a relevant occupational health concern in small-scale printing workplaces. The observed but imprecise association between reported benzol handling and elevated SPMA further suggests possible low-level benzene-related internal exposure and highlights the need to consider locally used chemical terminology and mixed solvent exposure when interpreting biomarker results. In settings where routine air monitoring is limited or unavailable, urinary biomonitoring may support both exposure surveillance and preventive occupational healthcare. To be most informative, biomonitoring findings should be interpreted together with workplace observations, product information, ventilation conditions, protective equipment use, and worker education practices, with the aim of directing health management resources toward small enterprises where solvent exposure warrants closer follow-up and targeted preventive action.

## Figures and Tables

**Table 1:** Demographic and occupational characteristics of study participants by urinary SPMA quartile.

Characteristics	Total (N = 21)	SPMA Q4 (n = 5)	SPMA Q1–3 (n = 16)	*p*
Age (years), mean ± SD	45.6 ± 14.0	53.0 ± 9.7	43.2 ± 14.5	0.11
Age group (years)				0.22
20–39	6 (28.6)	0 (0.0)	6 (37.5)	
40–59	11 (52.4)	3 (60.0)	8 (50.0)	
60+	4 (19.0)	2 (40.0)	2 (12.5)	
Nationality				0.53
Korean	17 (81.0)	5 (100.0)	12 (75.0)	
Foreign nationals	4 (19.0)	0 (0.0)	4 (25.0)	
Smoking status				0.46
Current	9 (42.9)	3 (60.0)	6 (37.5)	
Former	6 (28.6)	0 (0.0)	6 (37.5)	
Never	6 (28.6)	2 (40.0)	4 (25.0)	
Education				0.07 †
Middle school or less	4 (19.0)	0 (0.0)	4 (25.0)	
High school	13 (61.9)	2 (40.0)	11 (68.8)	
College	3 (14.3)	2 (40.0)	1 (6.2)	
Total work duration				0.22
<5 years	6 (28.6)	0 (0.0)	6 (37.5)	
5–9 years	5 (23.8)	1 (20.0)	4 (25.0)	
≥10 years	10 (47.6)	4 (80.0)	6 (37.5)	
Job type ‡				
Offset printing	15 (71.4)	4 (80.0)	11 (68.8)	1.00
UV printing	9 (42.9)	1 (20.0)	8 (50.0)	0.34

Data are presented as n (%) for categorical variables and mean ± standard deviation (SD) for continuous variables. *p*-values were calculated using Fisher’s exact test for categorical variables and the independent-samples *t*-test for continuous variables. *p* < 0.05; † *p* < 0.10, interpreted as suggestive evidence only in this exploratory analysis. ‡ Multiple responses were permitted. SPMA, S-phenylmercapturic acid; Q4, fourth quartile; SD, standard deviation.

**Table 2:** Urinary biomarker concentrations of benzene and toluene in the study participants (N = 21).

Biomarker	t,t-MA	SPMA	Phenol	o-Cresol
Exposure marker	Benzene	Benzene	Benzene	Toluene
GM (GSD)	94.2 (3.55)	2.3 (2.47)	4926.9 (2.22)	320.6 (2.33)
Median (range)	78.8 (9.4–2076.6)	1.8 (1.0–40.1)	4687.3 (1348–39,840)	239.9 (62.7–1549.1)
Quartile cutoff				
Q1	≤33.8	≤1.5	≤3157.8	≤185.5
Q2	≤78.8	≤1.8	≤4687.3	≤239.9
Q3	≤157.7	≤2.3	≤6328.4	≤618.6
Reference-value category				
Below 10% of reference value	7 (33.3)	17 (81.0)	12 (57.1)	0 (0.0)
10–99% of reference value	12 (57.1)	3 (14.3)	9 (42.9)	12 (57.1)
At or above reference value	2 (9.5)	1 (4.8)	0 (0.0)	9 (42.9)

All concentrations were creatinine-adjusted and expressed as μg/g creatinine. The ACGIH BEI values were used for t,t-MA (500 μg/g creatinine), SPMA (25 μg/g creatinine), and o-cresol (300 μg/g creatinine). The Korean biological reference value was used for phenol (50,000 μg/g creatinine). GM: geometric mean; GSD: geometric standard deviation; BEI: biological exposure index; SPMA: S-phenylmercapturic acid; t,t-MA: trans,trans-muconic acid.

**Table 3:** Association between self-reported chemical handling and elevated urinary biomarker levels (Q4), estimated using Firth penalized logistic regression.

Outcome	Variable	cOR (95% CI)	*p*	aOR (95% CI)	*p*
SPMA (Q4)	Age (per year)	1.05 (0.98–1.16)	0.197	-	-
	Benzol	6.27 (0.88–74.98)	0.068 †	6.04 (0.75–104.90)	0.093 †
	Toluene	0.42 (0.04–2.98)	0.399	0.53 (0.05–3.80)	0.537
t,t-MA (Q4)	Age (per year)	1.10 (1.01–1.29)	0.034 *	-	-
	Benzol	0.90 (0.12–6.00)	0.917	1.01 (0.12–9.76)	0.991
	Toluene	1.15 (0.15–7.78)	0.883	1.51 (0.17–14.22)	0.702
Phenol (Q4)	Age (per year)	1.06 (0.98–1.18)	0.160	-	-
	Benzol	0.90 (0.12–6.00)	0.917	1.10 (0.13–10.40)	0.926
	Toluene	1.15 (0.15–7.78)	0.883	1.28 (0.16–10.07)	0.805
o-Cresol (Q4)	Age (per year)	1.02 (0.95–1.11)	0.578	-	-
	Benzol	0.90 (0.12–6.00)	0.917	0.96 (0.12–7.70)	0.971
	Toluene	2.93 (0.44–22.71)	0.266	2.97 (0.45–23.67)	0.256

cOR, crude odds ratio; aOR, age-adjusted odds ratio; CI, confidence interval; Q4, fourth quartile. Age ORs are presented per 1-year increase. * *p* < 0.05; † *p* < 0.10, interpreted as suggestive evidence only in this exploratory analysis.

## Data Availability

The datasets generated and analyzed during the current study are available from the corresponding author upon reasonable request and subject to institutional and ethical restrictions.

## Supplementary Materials

The following supporting information can be downloaded at https://www.mdpi.com/article/10.3390/healthcare14131856/s1, Supplementary File S1: Standard questionnaire used for worker interviews; Supplementary Table S1: Summary of analytical methods and detection limits for urinary biomarkers.

## Abbreviations

The following abbreviations are used in this manuscript:

ACGIH	American Conference of Governmental Industrial Hygienists
aOR	adjusted odds ratio
BEI	biological exposure index
CI	confidence interval
cOR	crude odds ratio
CRM	certified reference material
EI	electron ionization
ESI	electrospray ionization
GM	geometric mean
GSD	geometric standard deviation
HS-GC-MS	headspace gas chromatography–mass spectrometry
IRB	Institutional Review Board
KODSC	Korea Occupational Disease Surveillance Center
KOSHA	Korea Occupational Safety and Health Agency
LC-MS/MS	liquid chromatography–tandem mass spectrometry
LOD	limit of detection
LOQ	limit of quantification
MRM	multiple reaction monitoring
OR	odds ratio
QC	quality control
SIM	selected ion monitoring
SPMA	S-phenylmercapturic acid
t,t-MA	trans,trans-muconic acid
TLV	threshold limit value
