# Supplementary material for: Biological Monitoring as a Preventive Occupational Healthcare Tool: Urinary Biomarkers of Benzene and Toluene Exposure Among Small-Scale Printing Workers in South Korea
Source: Healthcare (Basel). 2026 Jun 25;14(13):1856. doi: 10.3390/healthcare14131856 (PMC13362319; doi:10.3390/healthcare14131856)
Supplement: Supplementary file 1 [file healthcare-14-01856-s001.zip › 4370018_Supplementary File S1_260625_f.pdf]

[Supplementary File S1]

Health Risk Survey on Chemical Exposure among Printing Workers

English Translation of the Korean Questionnaire

2025

Seoul Occupational Disease Surveillance Center, Hanyang University Hospital

Original Korean title: 「인쇄업 종사자의 화학물질 노출에 따른 건강 위해 조사」

Health Risk Survey on Chemical Exposure among Printing Workers

Greetings.

This study is being conducted with support from the Occupational Health Standards Division of the Ministry of Employment and Labor. The fundamental purpose of this study is to investigate, follow up, and monitor the health status of workers and others in order to protect and promote their health, based on the operation of the occupational disease monitoring system under the Guidelines for Workers' Health Promotion Activities (Notice No. 2022-33), pursuant to the Occupational Safety and Health Act.

The printing industry is a traditional manufacturing sector, but it also has complex characteristics because it responds sensitively to the cultural industry. Since the COVID-19 pandemic, the digitalization of media and the expansion of platforms have increased demand for mass printing and high-quality output, leading to the reactivation of traditional offset printing.

Therefore, this study aims to explain occupational health risks in the printing industry through the concept of the “reemergence of old risks.” It also aims to investigate chemical exposure and current working conditions among printing workers and to provide evidence for improving the occupational work environment and health management measures.

Your responses will serve as valuable data for this study. We kindly ask you to respond honestly despite your busy schedule. Thank you for your cooperation.

September 16, 2025

- Survey implementing institution: Seoul Occupational Disease Surveillance Center, Hanyang University Hospital
- Contact person: Jungho Hwang / Yangwoo Kim, Seoul Occupational Disease Surveillance Center, Hanyang University Hospital (kodcseoul@hyumc.com)
- Principal investigator: Inah Kim, Department of Occupational and Environmental Medicine, Hanyang University College of Medicine; Seoul Occupational Disease Surveillance Center
- Contact: Tel. 02-6953-6718 / Fax 02-6953-6719

Statistics Act

Article 33 (Protection of Confidentiality)

1) Matters learned in the process of producing statistics that fall under the confidentiality of an individual, corporation, organization, or other entity shall be protected.

2) Confidential data collected from an individual, corporation, organization, or other entity for the production of statistics shall not be used for purposes other than the production of statistics.

LIST ID	Interviewer	Reviewer 1	Reviewer 2	Reviewer 3

To be completed after questionnaire collection.

Date of survey: 20____ / ____ / ____	Interviewer: (_______________)

Personal Information

The following questions concern your personal information. Please complete all items without omission.

Is your nationality Republic of Korea? [ ] 1. Yes (go to <1>) [ ] 2. No (go to <2>)

<1> Korean nationals

Name	(________________________)	Sex	[ ] 1. Male [ ] 2. Female
Resident registration number	__________ - __________		
Address	(_______________) Province/City (____________________) City/County/District		
Mobile phone number	____ - ____ - ____		

<2> Non-Korean nationals

Name	(________________________)	Sex	[ ] 1. Male [ ] 2. Female
Date of birth	____________________		
Mobile phone number	____ - ____ - ____		
What is your nationality?			
	[ ] 1. Vietnam<br>[ ] 2. China<br>[ ] 3. Indonesia	[ ] 4. Uzbekistan<br>[ ] 5. Cambodia	[ ] 6. Other country:<br>____________________

General Work Environment

The following questions concern your general work environment.

1. How long have you worked at printing workplaces in your lifetime?

[ ] 1. Less than 1 year	[ ] 2. 1-5 years
[ ] 3. 6-10 years	[ ] 4. 11-20 years
[ ] 5. More than 20 years	

2. How long have you worked at your current printing workplace?

[ ] 1. Less than 1 year	[ ] 2. 1-5 years
[ ] 3. 6-10 years	[ ] 4. 11-20 years
[ ] 5. More than 20 years	

3. Please check all types of work you currently perform at your printing workplace. *(Select all that apply.)*

[ ] 1. Order/manuscript handling	[ ] 2. Planning/design
[ ] 3. Editing/color work	[ ] 4. CTP (printing plate)
[ ] 5. Accounting/personnel administration	[ ] 6. Offset printing
[ ] 7. Silk-screen printing	[ ] 8. UV printing
[ ] 9. Clothing/textile printing	[ ] 10. Sticker/box printing
[ ] 11. Laminating/coating	[ ] 12. Thomson die-cutting
[ ] 13. Gold/silver foil stamping	[ ] 14. Logistics/transport
[ ] 15. Other: __________	

4. What is the one task you perform most often at your current printing workplace?

[ ] 1. Order/manuscript handling	[ ] 2. Planning/design
[ ] 3. Editing/color work	[ ] 4. CTP (printing plate)
[ ] 5. Accounting/personnel administration	[ ] 6. Offset printing
[ ] 7. Silk-screen printing	[ ] 8. UV printing
[ ] 9. Clothing/textile printing	[ ] 10. Sticker/box printing
[ ] 11. Laminating/coating	[ ] 12. Thomson die-cutting
[ ] 13. Gold/silver foil stamping	[ ] 14. Logistics/transport
[ ] 15. Other: __________	

5. During the past month, how many days per week did you usually come to your current workplace?

[ ] 1. Fewer than 3 days	[ ] 2. 4 days
[ ] 3. 5 days	[ ] 4. 6 days
[ ] 5. 7 days	

6. During the past month, how long did you usually stay at the workplace per day from arrival to leaving work?

[ ] 1. Less than 5 hours	[ ] 2. 6-7 hours
[ ] 3. 8-9 hours	[ ] 4. 10-11 hours
[ ] 5. 12 hours or more	

Chemical Exposure

The following questions concern chemical exposure at printing workplaces.

7. Do you use chemicals, inks/paints, thinners, sprays, or similar products at your current workplace?

[ ] 1. Yes (go to Q8)	[ ] 2. No (go to Q14)

8. Please write down all chemicals or products used at your current workplace, as many as you know.

Examples from the original Korean questionnaire: water, sol/cleaning solvent, thinner, ppangppangi solution, chemical, spray, WD, ttakttaki, Norumal, toluene, X, etc. Local product names and colloquial terms may be written as used at the workplace.

---

9. Among the chemicals or products listed above, which one do you use most often?

---

10. What is the purpose of the chemical or product you use most often?

[ ] 1. Cleaning agent (ppangppangi)	[ ] 2. Diluent/thinner
[ ] 3. Ink/paint	[ ] 4. Adhesive
[ ] 5. Other: ___________	

11. How many times per day do you usually use the chemical or product you use most often?

[ ] 1. Once	[ ] 2. 2-3 times
[ ] 3. 4-5 times	[ ] 4. 6-10 times
[ ] 5. More than 10 times	

12. How well do you know how harmful the chemical or product you currently use is to your body?

[ ] 1. Know very well	[ ] 2. Know somewhat
[ ] 3. Do not know well	[ ] 4. Do not know at all

13. Do you think the place where you mainly work is well ventilated?

[ ] 1. Very well ventilated	[ ] 2. Somewhat well ventilated
[ ] 3. Not well ventilated	[ ] 4. Not ventilated at all
[ ] 5. Do not know	

Symptoms and Health Status

The following questions concern symptoms and health conditions that occurred while working or after work.

14. Have you ever received a health examination (special health examination) because of your work at the current workplace?

[ ] 1. Yes	[ ] 2. No
[ ] 3. Do not know	

15. During the past month, please select all symptoms that occurred while working or after work. *(Select all that apply.)*

[ ] 1. Headache	[ ] 2. Dizziness
[ ] 3. Nausea	[ ] 4. Abdominal bloating/discomfort
[ ] 5. Vomiting	[ ] 6. Tinnitus
[ ] 7. Eye pain	[ ] 8. Cough
[ ] 9. Sputum/phlegm	[ ] 10. Chest tightness
[ ] 11. Lower back pain	[ ] 12. Shoulder/arm pain
[ ] 13. Leg/knee pain	[ ] 14. Weakness in hands or feet
[ ] 15. None	[ ] 16. Other: ______________________________

16. Have you ever received medical treatment or a health examination because of the symptoms checked above?

[ ] 1. Yes	[ ] 2. No

17. How do you perceive your current health status?

[ ] 1. Very good	[ ] 2. Good
[ ] 3. Fair	[ ] 4. Poor
[ ] 5. Very poor	

18. Are you taking medication for any of the following diseases? Please select all that apply. *(Select all that apply.)*

[ ] 1. Hypertension	[ ] 2. Diabetes mellitus
[ ] 3. Dyslipidemia	[ ] 4. None
[ ] 5. Other: ____________________	

19. Have you ever been diagnosed with and treated for any of the following diseases? Please select all that apply. *(Select all that apply.)*

[ ] 1. Cerebral hemorrhage or cerebral infarction	[ ] 2. Angina or myocardial infarction
[ ] 3. Asthma or lung disease	[ ] 4. Cancer
[ ] 5. None	[ ] 6. Other: ____________________

Lifestyle

The following questions concern your lifestyle habits.

20. Have you ever smoked cigarettes or electronic cigarettes in your lifetime? If yes, do you currently smoke?

[ ] 1. Current smoker	[ ] 2. Former smoker
[ ] 3. Lifelong non-smoker	

21. During the past month, how often did you drink alcohol per week?

[ ] 1. 0 days	[ ] 2. 1-2 days
[ ] 3. 3-4 days	[ ] 4. 5-6 days
[ ] 5. 7 days	

Economic Activity

The following questions concern your economic activity.

22. How many family members currently live with you?

[ ] 1. 1 person (living alone)	[ ] 2. 2 people
[ ] 3. 3 people	[ ] 4. 4 people
[ ] 5. 5 or more people	

23. What is your average monthly household income, including the income of all family members living with you?

[ ] 1. Less than KRW 1,000,000	[ ] 2. KRW 1,000,000-2,000,000
[ ] 3. KRW 2,000,000-3,000,000	[ ] 4. KRW 3,000,000-4,000,000
[ ] 5. KRW 4,000,000 or more	

24. What is your highest level of education?

[ ] 1. Elementary school graduate	[ ] 2. Middle school graduate
[ ] 3. High school graduate	[ ] 4. Junior college graduate
[ ] 5. University graduate	

Workplace

The following questions concern your current workplace.

25. How long has your current workplace been in operation? Include the actual period of operation since establishment, including any corporate registration changes.

[ ] 1. Less than 1 year	[ ] 2. 1-5 years
[ ] 3. 6-10 years	[ ] 4. 10-20 years
[ ] 5. More than 20 years	

26. What is the size of your current workplace?

[ ] 1. One-person workplace	[ ] 2. 2-4 workers
[ ] 3. 5-9 workers	[ ] 4. 10-49 workers
[ ] 5. 50 or more workers	

27. What ventilation methods are used at your current workplace? Please select all that apply. *(Select all that apply.)*

[ ] 1. Opening windows	[ ] 2. Ceiling air-conditioning/air handling system
[ ] 3. Local exhaust ventilation	[ ] 4. Duct/filter system
[ ] 5. None	[ ] 6. Other: ____________________

28. What protective equipment is used at your current workplace? Please select all that apply. *(Select all that apply.)*

[ ] 1. General mask	[ ] 2. Respirator for organic vapors/chemical cartridge respirator
[ ] 3. Noise earplugs	[ ] 4. Chemical-resistant gloves
[ ] 5. None	[ ] 6. Other: ____________________

29. How often does your current workplace conduct occupational safety and health education?

[ ] 1. Every quarter (every 4 months)	[ ] 2. Every half-year (every 6 months)
[ ] 3. Once a year	[ ] 4. Occasionally every few years
[ ] 5. Not conducted	

End of questionnaire.
