# Supplementary material for: Biological Monitoring as a Preventive Occupational Healthcare Tool: Urinary Biomarkers of Benzene and Toluene Exposure Among Small-Scale Printing Workers in South Korea
Source: Healthcare (Basel). 2026 Jun 25;14(13):1856. doi: 10.3390/healthcare14131856 (PMC13362319; doi:10.3390/healthcare14131856)
Supplement: Supplementary file 1 [file healthcare-14-01856-s001.zip › 4370018_supplementary Table S1_260625_f.pdf]

[Supplementary Table S1]

Supplementary Table S1. Summary of analytical methods and detection limits for urinary biomarkers.

| Biomarker | Analytical instrument | Sample preparation and quantification | Internal standard / QC | LOD | LOQ |
|---|---|---|---|---|---|
| t,t-MA | LC-MS/MS, AB Sciex 5500 Plus | Urine sample pretreated with acetic acid/acetonitrile solution; ACE Excel 2 C18-AR column; ESI; MRM mode | t,t-MA-d4; NIST SRM 3673 accuracy 100.4% | 0.580 µg/L | 1.914 µg/L |
| SPMA | LC-MS/MS, AB Sciex 5500 Plus | Urine sample pretreated with acetic acid/acetonitrile solution; ACE Excel 2 C18-AR column; ESI; MRM mode | SPMA-d5; NIST SRM 3673 accuracy 94.4% | 0.030 µg/L | 0.099 µg/L |
| Phenol | HS-GC-MS, PerkinElmer Clarus 680-SQ8T | Urine sample prepared with sodium hydrogen sulfate and saturated sodium sulfate solution; DB-1701 column; EI; SIM mode | Phenol-d5; ClinChek 8924 accuracy 93.3% | 0.066 mg/L | 0.218 mg/L |
| o-Cresol | HS-GC-MS, PerkinElmer Clarus 680-SQ8T | Urine sample prepared with sodium hydrogen sulfate and saturated sodium sulfate solution; DB-1701 column; EI; SIM mode | Phenol-d5; ClinChek 8924 accuracy 103.2% | 0.006 mg/L | 0.020 mg/L |
| Creatinine | Kinetic colorimetric assay | Urinary creatinine was measured using the Jaffe kinetic colorimetric method and used for creatinine correction | Laboratory internal QC | — | — |

Abbreviations: EI, electron ionization; ESI, electrospray ionization; HS-GC-MS, headspace gas chromatography–mass spectrometry; LC-MS/MS, liquid chromatography–tandem mass spectrometry; LOD, limit of detection; LOQ, limit of quantification; MRM, multiple reaction monitoring; QC, quality control; SIM, selected ion monitoring; SPMA, S-phenylmercapturic acid; t,t-MA, trans,trans-muconic acid.
